# Time trends and modifiable factors of COVID-19 contact tracing coverage, Geneva, Switzerland, June 2020 to February 2022

**DOI:** 10.2807/1560-7917.ES.2024.29.3.2300228

**Published:** 2024-01-18

**Authors:** Denis Mongin, Nils Bürgisser, the Covid-SMC Study Group, Delphine Sophie Courvoisier, The Covid-SMC Study Group, Lucienne Da Silva Mora, Diem-Lan Vu, Lena Després, Rachel Dudouit, Béatrice Hirsch, Barbara Müller, Charlotte Roux, Géraldine Duc, Caroline Zahnd, Adriana Uribe Caparros, Guillaume Schimmel, Jean-Luc Falcone, Nuno M Silva, Thomas Goeury, Christophe Charpilloz, Silas Adamou, Pauline Brindel, Roberta Petrucci, Andrea Allgöwer, Abdel Kadjangaba, Christopher Abo Loha, Emilie Macher, Marc Vassant, Nadia Donnat, Philippe Pittet, Dominique Joubert, Samia Carballido, Ariane Germain, Sophie Bontemps, Elisabeth Delaporte, Camille Genecand, Aliki Metsini, Valérie Creac’h, Virginie Calatraba, Laura Flüeli, Hippolyte Piccard, Dan Lebowitz, Aglaé Tardin, Simon Regard

**Affiliations:** 1 Faculty of Medicine, University of Geneva, Geneva, Switzerland; 2 General internal medicine division, Department of Medicine, Geneva University Hospitals, Geneva, Switzerland; 3 The members of the Covid-SMC Study Group are listed under Collaborators; 4 Division Quality of care, Faculty of Medicine, University of Geneva, Geneva, Switzerland

**Keywords:** contact tracing, non-pharmaceutical interventions, COVID-19

## Abstract

**Background:**

Contact tracing was one of the central non-pharmaceutical interventions implemented worldwide to control the spread of SARS-CoV-2, but its effectiveness depends on its ability to detect contacts.

**Aim:**

Evaluate the proportion of secondary infections captured by the contact tracing system in Geneva.

**Methods:**

We analysed 166,892 concomitant infections occurring at the same given address from June 2020 until February 2022 using an extensive operational database of SARS-CoV-2 tests in Geneva. We used permutation to compare the total number of secondary infections occurring at the same address with that reported through manual contact tracing.

**Results:**

Contact tracing captured on average 41% of secondary infections, varying from 23% during epidemic peaks to 60% during low epidemic activity. People living in wealthy neighbourhoods were less likely to report contacts (odds ratio (OR): 1.6). People living in apartment buildings were also less likely to report contacts than those living in a house (OR: 1.1–3.1) depending on the SARS-CoV-2 variant, the building size and the presence of shops. This under-reporting of contacts in apartment buildings decreased during periods of mandatory wearing of face masks and restrictions on private gatherings.

**Conclusion:**

Contact tracing alone did not detect sufficient secondary infections to reduce the spread of SARS-CoV-2. Campaigns targeting specific populations, such as those in wealthy areas or apartment buildings, could enhance coverage. Additionally, measures like wearing face masks, improving ventilation and implementing restrictions on gatherings should also be considered to reduce infections resulting from interactions that may not be perceived as high risk.

**Key public health message**

**What did you want to address in this study and why?**

During the COVID-19 pandemic, contact tracing emerged as a key tool to control the spread of SARS-CoV-2. Its effectiveness is closely tied to its ability to identify contacts. We wanted to estimate how many infected people were detected by contact tracing, specifically, among people infected by another person living at the same address, and identify the factors associated with reporting contacts.

**What have we learnt from this study?**

On average, only 41% of people infected by someone living at the same address were reported by the person who infected them. Infected contacts were less likely to be reported by people in wealthy neighbourhoods, or who lived in apartment buildings with shops, except during periods with gathering restrictions and mandatory mask-wearing.

**What are the implications of your findings for public health?**

Our findings suggest that contact tracing alone was insufficient to prevent the spread of SARS-CoV-2. Contact tracing could be improved by targeting populations with high under-reporting and should be complemented by public health policies such as masking, air cleaning and clearing, or gathering restrictions to reduce the number of unnoticed infections.

## Introduction

In response to the COVID-19 pandemic in early 2020, many governments around the world implemented a large panel of measures, including non-pharmaceutical interventions (NPI) [1,2] to try to reduce the spread of severe acute respiratory syndrome coronavirus 2 (SARS-CoV-2). These interventions included lockdowns, contact tracing, travel restrictions, school or public building closures and bans on large events, all of which had varying effect on disease transmission [1].

Among the NPI, contact tracing rapidly became a key method of limiting virus transmission [3]. The idea of contact tracing is to reduce the onward spread of the virus by people who have been in contact with an infected index case, by limiting their ability to potentially infect others [4] through reducing social interaction and increasing protective measures (face masks, room ventilation). Contact tracing can be manual, semi-automated after a positive test result, or based on a mobile app. It can be initiated by health authorities (state-initiated), or by individuals (citizen-initiated). Finally, contact tracing can be extended forward to find contacts of the index case that can become infected, or backward to look for the contacts that contaminated the index case. Although theoretically effective [5], backward contact tracing was of limited use during the COVID-19 pandemic [6].

Forward contact tracing is highly effective only if all contacts are identified (by the index case or by an app), notified before they become contagious [7] and comply with protective measures (quarantine, face masks). In real-world settings, however, the true effectiveness of contact tracing for SARS-CoV-2 is estimated to range from a 63% reduction in new infections to no discernible difference [8] depending on the study and country involved. Contact tracing apps for SARS-CoV-2 have received much attention during the COVID-19 pandemic [9]. They were developed in different countries [10] and were shown, in controlled settings, to potentially have a large effect on reducing the virus spread [11]. Nevertheless, ecological studies reported varying effectiveness, from 45%, upon proper uptake and adherence [11], to very low ranging from 0.1–11% of additional infections detected by digital contact tracing alone [12].

There are several reasons for the relatively low effectiveness of contact tracing during the pandemic. First, contacts may not follow local recommendations, for instance they may evade quarantine or not use protective measures such as face masks. Second, the delay in notification and the number of contacts identified [13] may limit its effectiveness, since each new contact requires a minimum amount of time to be reached [14] and not all contacts can be reached in time to apply effective measures against the spread of SARS-CoV-2. Third, there could be intentional or un-intentional under-reporting. In other words, an index case may intentionally not declare contacts, or they could simply not be aware of being in contact with someone. Indeed, airborne transmission is the main contamination route of SARS-CoV-2 [15,16], and multiple examples of contamination across enclosed spaces without direct encounters between index and contact cases have been reported, such as contamination in corridors [17], shared spaces [18], via room ventilation systems [19] or even an air leak through a roof [20].

The aim of this study is to estimate the number of secondary infections captured by contact tracing, occurring at the same address as an index case, and identify factors associated with their detection.

## Methods

We used permutation statistics on more than 142,000 reported infections to estimate the number of secondary infections occurring at the same address as an index case. Using contact information provided by index cases, we estimated the number of infections that had been declared as contacts and assessed their association with demographic and socioeconomic characteristics.

### Data

We used all registered tests performed by persons living in the state of Geneva, Switzerland, from the Actionable Register of Geneva Outpatients and Inpatients with SARS-CoV-2 (ARGOS) register [21], which is an ongoing operational COVID-19 database created by the Geneva Directorate of Health. The register contains sociodemographic details, baseline and follow-up COVID-19-related health indicators and contact information.

### Setting and study period and

Geneva is a mainly urban state of 511,921 inhabitants as by the last census in December 2021, and with a high population density of 13,000 inhabitants per square kilometre. It is divided geographically into 417 administrative neighbourhoods (sous-secteurs) with a median population of around 1,000 persons. Each address in the dataset was geocoded using an exhaustive list of addresses in the State of Geneva, and each neighbourhood area was associated with a socioeconomic indicator provided by the Center for Territorial Analysis of Inequalities (hereafter the CATI-index) ranging from 0 (wealthiest) to 6 (poorest). This index was then divided into four further categories, similar to a previous study [22] (see details in the Supplementary material).

We used all data from 1 June 2020 to 1 February 2022 that included an address (3.4% of the reported infections did not have an address). As the data from the ARGOS register did not contain information about the SARS-CoV-2 variant type, we divided the study into periods of predominance of SARS-CoV-2 variants. Similar to another study based on the same data [23], we modelled the evolution of variants based on the data provided by Covariants [24] and the Global Initiative on Sharing Avian Influenza Data [25] in the Geneva region and defined the periods when respective variants were above 50% of all circulating variants (Box).

Box **Periods defined by dominant SARS-CoV-2 variant circulation for the study**

- 1 June 2020–5 January 2021: SARS-CoV-2 EU variants (Pango lineage designation B.1.177 (EU1) and B.1.620 (EU2))
- 6 January 2021–14 June 2021: SARS-CoV-2 Alpha variant (Pango lineage designation B.1.1.7)
- 15 June 2021–17 December 2021: SARS-CoV-2 Delta variant (Pango lineage designation B.1.617.2)
- 18 December 2021– 1 February 2022: SARS-CoV-2 Omicron variant (Pango lineage designation B.1.1.529, mainly variant BA.1 circulating)

PANGO: Phylogenetic Assignment of Named Global Outbreak.

Details about the calculations, the different NPIs in place during these periods and the vaccine used in Geneva have previously been reported [23] and are available in the Supplementary material.

### Definition, declaration and follow up of contacts

In Geneva, every person who tested positive for SARS-CoV-2 was legally obligated to declare their contacts. Contacts were defined by the Swiss Confederation as persons having had an interaction with the infected person for a duration of at least 15 minutes at a distance of less than 1.5 m, up to 48 hours before the index case’s symptoms, or in absence of symptoms, up to 5 days after the index case’s positive test.

After contact tracing was implemented in Geneva on 27 April 2020, declared contacts were obligated to quarantine for 10 days. Children under 12 years were exempt. From 8 February 2021, declared contacts could shorten their quarantine by providing a negative nasopharyngeal or oropharyngeal SARS-CoV-2 PCR test on day 7. Quarantine was shortened to 7 days on 31 December 2021 and to 5 days on 12 January 2022. By the end of 2021, vaccinated persons (at least 2 doses of mRNA vaccine i.e. Moderna mRNA-1273 or Pfizer BNT162b2, or one dose of Janssen Ad26.COV2-S vaccine) or persons with a positive SARS-CoV-2 test within the last 4 months were not obligated to quarantine after contact with an infected case. From October 2020, health professionals were allowed to work even if they were contacts of an infected person. However, they were systematically tested by their institution, and if tested positive, had to isolate for at least 48 hours. They could go back to work after 48 hours if they only had mild symptoms and no fever, while pursuing barrier measures (wearing face masks, eating alone) during at least 7 days when caring for patients. A graphical timeline is provided in the Supplementary material to summarise the changes in quarantine rules.

In January 2021, an anthropologist was hired by the Geneva Directorate of Health, who trained the contact tracing team in motivational interviewing and performed a field study to understand the barriers and facilitating factors to declaring one’s contacts.

Contact information was initially collected by telephone interviews of index cases (February 2020 to end of April 2020). From May 2020, index cases could provide their contacts’ names and phone numbers via an online form and information was completed when the index case was called. Contacts were sent a message telling them they were a contact and should quarantine, and were then contacted by phone. Additionally, an online form was implemented at the end of September 2020 to support the phone calls, where the contacts could complete the required information themselves. From mid-December 2021, the phone calls could not be maintained due to the high number of cases. Therefore, contact information was only obtained from the online forms.

### Outcomes: secondary infections occurring at the same address and absence of reporting

Coverage of contact tracing can be estimated by dividing the number of infections captured by the contact tracing by the total number of infections recorded. Although simple, this method has two caveats. First, Geneva is a region that shares its border with France. As a result, its population is increasing due to workers commuting between the two countries. France has its own contact tracing system, which makes the number of secondary infections of French citizens working in Geneva difficult to estimate. Secondly, this calculation does not allow us to consider modifiable factors such as socioeconomic or living conditions. We thus decided to restrict our study to secondary infections occurring at the same address as an index case, in Geneva, which will be our primary outcome.

To estimate contact tracing coverage, we first identified concurrent infections of two persons living at the same address and having a positive SARS-CoV-2 test result less than 10 days apart. The date associated with the concurrent infection was the middle date between the two test results. We then used the exhaustive list of declared contacts to define the binary variable ‘absence of reporting’ as being 0 if the concurrent infection was captured by the contact tracing and 1 otherwise. We considered the possibility that the concurrent infection had been declared either by the index case or the contact and did not restrict to any specific form of relationship type declared by the index case.

Concurrent infections capture both infections that are related due to living at the same address (secondary infections), and infections that occur by chance at the same address (concomitant infections, i.e. two persons living at the same address can be infected 10 days apart by other persons elsewhere).

N_concurrent_ = N_secondary_ + N_concomitant_

In order to determine the number of secondary infections, we first estimated the number of concomitant infections corresponding to the null hypothesis that there are no excess infections due to living at the same address (H0: N_secondary_ = 0). In other words, under this null hypothesis, concurrent infections occurring at the same address are due to chance only (N_concurrent_ = N_concomitant_). Permutation techniques can be used to estimate the frequency of concurrent infections under the null hypothesis [26]. This consists of permuting randomly (sampling without replacement) each person’s address and then computing the number of concurrent infections at the same address. This gives us the number of concomitant infections, that is the number of infections occurring at the same address only by chance (because the addresses were permuted). We then estimated the number of secondary infections occurring at a given address as the difference between the raw number of concurrent infections at a given address, obtained from the ARGOS register, and the ones obtained by permutation. We performed 1,000 permutations and operationalised the estimation of secondary infections as the median value of the difference obtained. To account for potential confounding, addresses were permuted within each neighbourhood and within each type of residential building. Permuting within each type of neighbourhood allows us to avoid confounding caused by the socioeconomic condition of the neighbourhood or by shared services, such as schools, grocery stores and some public transportation. Permuting within each residential building type allows us to avoid confounding caused by the association between concomitant infections and the size of the building. Indeed, the probability of having a concurrent infection by chance for two persons living at the same address is higher in a large building than in a small house.

### Statistical analysis

Descriptive statistics were provided with counts and proportions for categorical variables, and with median and inter quartile ranges (IQR) for continuous variables.

For secondary infections occurring at the same address, 95% confidence intervals (CI) were operationalised as the 2.5% and 97.5% quantile of the difference between the number of concurrent infections at a given address and the concomitant infections obtained by permutation. This analysis was stratified by SARS-CoV-2 variants.

To examine the association of gender, vaccination, living characteristics and socioeconomic characteristics with potential under-reporting of contacts, we calculated their odd ratios. To do so we applied for each permutation a generalised linear model using the absence of reporting as outcome, with CATI-index, type of residential building, number of people living at the address, vaccination status of the two persons of the concurrent infection dyads and their gender as covariates and ‘absence of reporting’ as the independent variable. Vaccination status was recalculated for each permutation at the date of corresponding concurrent infection. The final estimates of the model were given by the median and 2.5% and 97.5% quantiles of the differences between the estimates obtained for the raw dataset and the ones obtained for each of the permuted datasets.

All analyses were performed using R software version 4.1.0 [27], and the University of Geneva high-performance computing cluster Baobab.

### Covariates

Vaccination status was operationalised as fully vaccinated if both persons were vaccinated with at least one dose, mixed if one of the two was vaccinated with at least one dose and not vaccinated if neither were vaccinated. Vaccination status was calculated at the date of secondary infection. Sex was operationalised as male if both persons were male, female if both were female and mixed otherwise.

As contact tracing coverage may be influenced by the number of social interactions and the environment of the encounter, we categorised the type of dwelling in terms of both the population living at the address and the type of residential building. We thus decided to consider addresses where two or fewer people lived, separately from larger households or apartment buildings, since they were less likely to have social interaction with people living with them. We also distinguished between residential buildings containing shops, and ones without. The type of building was therefore operationalised into six categories (detailed explanation in the Supplementary material), in buildings with up to 2 inhabitants: (i) houses with isolated persons, in buildings with more than 2 inhabitants: (ii) houses (family houses); (iii) buildings with no shops and less than 40 inhabitants; (iv) buildings with no shops and more than 40 inhabitants; (v) building with shops and less than 40 inhabitants; (vi) building with shops and more than 40 inhabitants.

Of note, in the Geneva region, buildings in the city often have shops on the ground floor unlike buildings in more rural areas.

### Sensitivity analysis

The delay between two positive tests at the same address used to define concurrent infections was set in the main analysis as twice 5 days - the mean incubation period of early variants [28]. As this delay could influence our results, we performed two sensitivity analyses by defining concurrent infections with a shorter delay of 6 days (twice 3 days) or 14 days (twice 7 days) between the two positive tests.

## Results

Over the period of this study, 25,297 addresses had reported at least two persons with a positive SARS-CoV-2 test result less than 10 days apart (i.e. at least one concurrent infection, Table 1). The median number of concurrent infection dyads at these addresses was 3 (inter quartile range (IQR): 1–6), although it was lowest during the Alpha wave (median: 1, IQR: 1–3) and highest during the Omicron wave (median: 3, IQR: 1–10). The addresses were mainly situated in the wealthiest (37%) and poorest areas (30%) and concerned a median of 29 (IQR: 13–47) persons, with no notable change across time. The main type of residential building were buildings with no shops and less than 40 inhabitants (32%), followed by buildings with shops and less than 40 inhabitants (17%), family houses (16%), buildings with shops and more than 40 inhabitants (13%) and houses with maximum 2 persons (3%).

**Table 1:** Characteristics of the addresses where at least one concurrent infection occurred, during the overall period (overall), stratified by SARS-CoV-2 variant, Geneva, Switzerland, June 2020–February 2022 (n = 25,297)

Address characteristics	Overall		SARS-CoV-2 variant							
			EU		Alpha		Delta		Omicron	
Number of addresses	25,196		6,596		2,805		5,007		10,788	
Median number of concurrent infections per address (IQR)	3.00 (1.00–6.00)		2.00 (1.00–4.00)		1.00 (1.00–3.00)		2.00 (1.00–4.00)		3.00 (1.00–10.00)	
CATI-index	n	%	n	%	n	%	n	%	n	%
0	9,238	37.0	2,334	35.6	936	33.6	1,881	38.0	4,087	38.3
1	3,939	15.8	992	15.1	476	17.1	773	15.6	1,698	15.9
2–3	4,419	17.7	1,206	18.4	496	17.8	844	17.0	1,873	17.6
4–6	7,360	29.5	2,027	30.9	874	31.4	1,456	29.4	3,003	28.2
Building type	n	%	n	%	n	%	n	%	n	%
Family houses (reference)	4,010	15.9	873	13.2	397	14.1	838	16.7	1,902	17.6
House with isolated persons	726	2.9	250	3.8	79	2.8	108	2.1	289	2.7
Building without shops less than 40 inhabitants	8,076	31.9	2,021	30.5	826	29.4	1,548	30.8	3,681	34.0
Building without shops more than 40 inhabitants	5,029	19.9	1,444	21.8	687	24.4	1,075	21.4	1,823	16.8
Building with shops less than 40 inhabitants	4,256	16.8	1,14	17.3	406	14.4	757	15.0	1,951	18.0
Building with shops more than 40 inhabitants	3,200	12.6	888	13.4	417	14.8	705	14.0	1,190	11.0

CATI: Center for Territorial Analysis of Inequalities; IQR: interquartile range.

CATI-index is the socioeconomic index of the neighbourhood, with 0 the wealthiest and 6 the poorest.

### Excess concurrent infections

During the period of interest, 166,892 raw concurrent infections occurred (Table 2). The null hypothesis estimation yielded 117,617 (95% CI: 116,363–118,945) concurrent infections. The estimated excess number of concurrent infections occurring at the same address was 49,275 (95% CI: 47,947–50,529).

**Table 2:** Number of persons infected at the same address 10 days apart, in the ARGOS register and when permuting addresses, Geneva, Switzerland, June 2020–February 2022 (n = 25,297 addresses)

Infection and contagion characteristics at the same address	Total	SARS-CoV-2 variant			
		EU	Alpha	Delta	Omicron
Number of raw concurrent infections	166,892	38,562	9,551	19,382	99,397
Number of concurrent infections in permutations (95% CI)	117,617 (118,945–116,363)	22,722 (23,041–22,412)	2,484 (2,573–2,398)	9,499 (9,714–9,306)	82,912 (83,617–82,247)
Estimated contagions at the address (95% CI)	49,275 (47,947–50,529)	15,840 (15,521–16,150)	7,067 (6,978–7,153)	9,883 (9,668–10,076)	16,485 (15,780–17,150)
Number of contacts declared living at the same address	20,990	5,341	3,687	5,085	6,877
Percentage of contagions declared (95% CI)	42.6% (41.5–43.8)	33.7% (33.1–34.4)	52.2% (51.5–52.8)	51.5% (50.5–52.6)	41.7% (40.1–43.6)

CI: confidence interval.

Estimations for permutation are the median of 1,000 permutations given with their percentile confidence intervals (2.5%–97.5% range).

### Proportion of infections reported through contact tracing

Declared contacts (n = 20,990), living at the same address as their index case and who became positive less than 10 days following the index case’s test result accounted for 42.6% (95% CI:41.5–43.8) of the estimated concurrent infections. This percentage was at its lowest during the EU variant wave with 33.7% (95% CI: 33.1–34.4), rose above 50% during the Alpha and Delta waves (52.2%, 95% CI: 51.5–52.8 and 51.4%, 95% CI: 50.4–52.6, respectively) and decreased to 41.8% (95% CI: 40.0–43.6) during the Omicron wave.

The monthly evolution of this percentage of infections captured by contact tracing fluctuated between 67% and 23% (Figure) and tended to be lower when the number of COVID-19 cases was high. The lowest numbers of declared contacts reported were observed during the two periods where there were more than 10,000 COVID-19 cases per month (the peak of the EU wave and the end of the Delta/start of the Omicron wave). Of note, the strongest increase of the rate of declared contacts reported was in January 2021, increasing from 23% to 50%.

**Figure:**
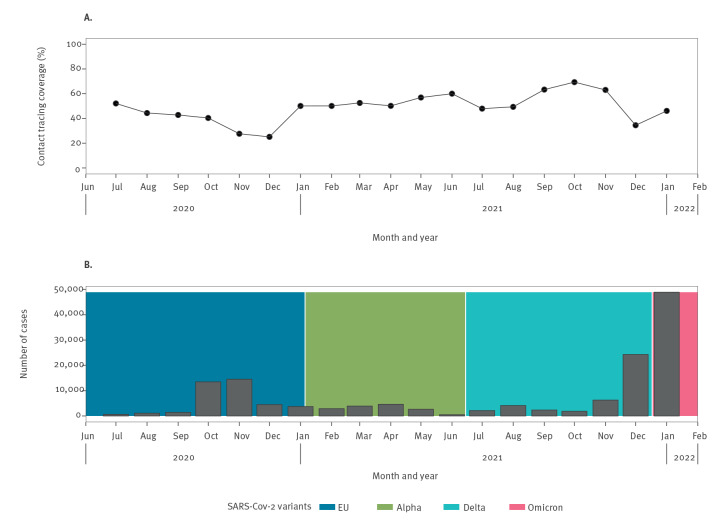
Monthly evolution of contact tracing coverage (A) and of the number of positive COVID-19 tests in Geneva (B)

### Determinants of absence of reporting

When compared with the adult age category (17–65 years old), a contact child (age under 17) tended to have more chance of being under-reported during early SARS-CoV-2 variants. During the EU and Omicron waves, an index case younger than 17 was less likely to have their contacts reported (odds ratio (OR): 1.27, 95% CI: 1.07–1.56 during Omicron variant circulation).

The socioeconomic status of the neighbourhood had a strong ‘dose-response’ association with under-reporting: persons from the poorest neighbourhood were less likely to under-declare their contacts, with an OR of 0.59 (95% CI: 0.47–0.72) for the most disadvantaged neighbourhood during the Omicron wave (Table 3).

**Table 3:** Results of the multivariable generalised model for under-reporting of the secondary SARS-CoV-2 infections, Geneva, Switzerland, June 2020–February 2022 (n = 25,297)

Variable	SARS-CoV-2 variant							
	EU		Alpha		Delta		Omicron	
	OR	95% CI	OR	95% CI	OR	95% CI	OR	95% CI
Contact age in years (reference: 17–65)								
0–16	1.25	0.91–1.65	1.16	0.91–1.5	1.17	0.97–1.42	1.09	0.89–1.31
≥ 65	0.94	0.62–1.31	0.9	0.39–1.6	0.87	0.44–1.36	1.12	0.69–1.68
Index age in years (reference: 17–65)								
0–16	1.14	0.78–1.53	1.23	0.91–1.64	0.98	0.8–1.18	1.27	1.07–1.56
≥ 65	0.95	0.63–1.3	0.9	0.37–1.58	0.88	0.46–1.4	0.71	0.32–1.18
CATI-index (reference: 0)								
1	0.9	0.69–1.15	0.8	0.57–1.08	0.88	0.69–1.14	0.83	0.67–1.04
2–3	**0.68**	**0.5–0.88**	0.86	0.62–1.16	0.87	0.69–1.14	**0.77**	**0.62–0.98**
4–6	**0.64**	**0.5–0.81**	**0.65**	**0.48–0.86**	**0.66**	**0.51–0.84**	**0.59**	**0.47–0.72**
Sex (reference: male-male)								
Female-female	1.17	0.84–1.76	0.97	0.68–1.32	**1.34**	**1.02–1.85**	1.06	0.81–1.36
Mixed	1.22	0.9–1.68	1.12	0.83–1.49	1.22	0.92–1.58	1.06	0.86–1.31
Type of building (single house)								
House with isolated persons	1.94	0.77–4.14	1.52	0.47–5.33	**3.56**	**1.2–9.2**	**2.26**	**1.02–4.33**
Building without shops less than 40 inhabitants	1.12	0.74–1.78	0.77	0.41–1.46	1.12	0.72–1.76	1.17	0.82–1.69
Building without shops more than 40 inhabitants	**1.79**	**1.15–2.99**	1.03	0.59–1.82	**1.68**	**1.12–2.6**	**2.05**	**1.45–3.03**
Building with shops less than 40 inhabitants	**1.85**	**1.17–2.93**	1.24	0.66–2.23	**1.94**	**1.2–3.12**	**2.38**	**1.66–3.47**
Building with shops more than 40 inhabitants	**2.57**	**1.65–4.04**	1.08	0.61–1.99	**2.04**	**1.3–3.27**	**3.13**	**2.19–4.66**
Vaccination status (both not vaccinated)								
Index: not vaccinated; Contact: vaccinated	NA		0.66	0.2–1.39	0.86	0.64–1.13	0.83	0.65–1.06
Index: vaccinated; Contact: not vaccinated			0.64	0.21–1.31	**0.7**	**0.51–0.91**	0.84	0.66–1.05
Index: vaccinated; Contact: vaccinated			1.32	0–5.23	0.97	0.68–1.35	0.9	0.71–1.13

CATI: Center for Territorial Analysis of Inequalities; CI: confidence interval; NA: not applicable; OR: odds ratio.

OR with a 95% confidence interval not encompassing 1 are presented in bold.

During the EU, Delta and Omicron waves, under-reporting was found to be significantly affected by type of residential building. When compared with family houses, under-reporting increased with the number of inhabitants in buildings and with the presence of shops. We observed no significant effect for buildings without shops and less than 40 inhabitants. However, we found the OR of not reporting contacts in buildings without shops with more than 40 inhabitants ranged from 1.79 (95% CI: 1.15–2.99) during the EU wave, to 2.06, (95% CI: 1.46–3.03) during the Omicron wave, and the odds ratio of not reporting contacts in buildings with shops and less than 40 inhabitants from 1.85 (95% CI: 1.17–2.93) during the EU wave, to 2.38, (95% CI: 1.66–3.47) during the Omicron wave. The highest OR was found during the EU and Omicron waves for buildings with shops and more than 40 inhabitants (OR: 2.57, 95% CI: 1.65–4.04 and OR: 3.13, 95% CI: 2.19–4.66, respectively). Houses with isolated persons had significantly higher OR of not reporting during the Delta and Omicron waves, but with a very large confidence interval (OR: 3.56, 95% CI: 1.20–9.20 and OR: 2.26, 95% CI: 1.02–4.33, respectively).

Being vaccinated increased the odds of declaring contacts only when one of the two persons implied was vaccinated. This effect reached significance during the Delta wave when the index case was vaccinated and the contact was not (OR: 0.70, 95% CI: 0.51–0.91) and during the Omicron wave when the contact was vaccinated and the index case not (OR: 0.84, 95% CI: 0.66–1.05).

### Sensitivity analysis

Considering 6 days between two positive tests at the same address to define concurrent infections yielded a global contact coverage of 40.1%, 95% CI: 39.3–41.1 (48,895 estimated secondary infections occurring at the address, 95% CI: 47,300–50,400, for 21,733 reported contacts). Considering 14 days between two positive tests yielded a 44.4%, 95% CI: 43.1–45.9 contact coverage (44,875 estimated secondary infections occurring at the address 95% CI: 43,861–45,859, for 18,017 reported contacts). The determinant of absence of reporting provided OR very similar to those of the main analysis. Detailed results for the two sensitivity analyses can be found in the Supplementary material.

## Discussion

The complete database of COVID-19 infections occurring in Geneva over a period of almost 2 years allowed us to estimate the capacity of contact tracing to capture infectious contacts occurring at the same living address as their index case. In this study, on average, contact tracing allowed to detect 41% of secondary infections occurring at the same given address. This percentage varied over time and was lower during the last months of 2020 and at the beginning of the Omicron wave in December 2021. The principal determinants of absence of reporting contacts were living in a wealthy neighbourhood, younger age and living in a populated building with shops. Mixed vaccination status (one individual vaccinated, the other not) was associated with higher reporting.

Contact tracing can only have a sustained meaningful effect on disease transmission for variants with relatively low effective reproduction numbers, as long as the coverage of contact is high and the delay in notifying contacts remains short. Indeed, a simulation study with isolation of cases only [14] showed that detecting only 40% of contacts can control more than 80% of outbreaks, but only if the reproductive number is low. However, if the effective reproduction number is 3.5, a contact coverage of 40% would not control more than 10% of outbreaks. For such reproduction number, controlling more than 80% of outbreaks would require a contact coverage of almost 90%. Modelling studies [7,29] considering low basic reproduction numbers estimated that the effect of contact tracing would start to have a real impact on the reproduction number if more than 50% of contacts were reached. Another study [30] showed that reducing contact tracing coverage from 80% to 40% would at least double the probability of a large outbreak even with few cases. Given that the basic reproduction number of Alpha, Delta and Omicron variants is above 3 and close to 8 for the latter [31,32], and that the proportion of contacts traced decreased during high viral activity periods, the impact of the manual contact tracing on the spread of COVID-19 may have been rather limited after the first two waves [33].

Several mechanisms can contribute to low coverage of contact tracing. The first one is the saturation of contact tracing capacity due to limited number of personnel and resources required to perform the contact tracing. A second one is intentional under-reporting, encompassing contacts not declared to avoid quarantine measures [34], but also contacts not declared because they were exempt from quarantine such as health professionals, vaccinated persons or children under 12 years. A third mechanism could be non-intentional unreported contacts, following infectious encounters that are not perceived as such, for example using an elevator after an infected person, walking past an infected neighbour at the shop, being infected by the aerosols escaping an infected neighbour’s apartment [17].

Our study shows indications of these three mechanisms. The effect of contact tracing capacity is evidenced in our study by a decrease in the percentage of contacts reported during periods with a high number of reported cases, reaching as low as 20%. The increase in contacts reported in January 2021 seems to correspond with implementation of guidelines by the Geneva Directorate of Health to encourage infected persons to declare their contacts during the phone interviews. Also during this month, the following measures were implemented from 18 January, and then gradually relaxed between May and June 2021: mandatory working remotely if possible, mandatory wearing of face masks at work and in shops, closure of shops not selling consumer essentials and public and private gatherings restricted to a maximum of five people. These public health measures could have helped people recognise possible contacts and declare them accordingly.

The findings of this study also suggest intentional under-reporting. For instance, the tendency to under-report children before the Omicron wave was consistent with the exemption of children from quarantine at the beginning of the pandemic in Geneva. Erroneous public health messages at the beginning of the pandemic could have helped foster under-reporting of children. Indeed, during the first COVID-19 wave in April 2020, a statement from the Head of Communicable Diseases Division at the Federal Office of Public Health in Switzerland, widely reported in news outlets, stated that infection in children was very unlikely and that transmission among children was close to none [35]. A second indication of intentional under-reporting was the increase of the odds of not declaring contacts increased with the wealth of the neighbourhood. This result may stem from the fact that persons living in wealthy neighbourhoods may have jobs allowing them to work remotely and having therefore a lower need of official quarantine certificates. It is also in line with studies that found that individuals from higher social class exhibited greater unethical decision-making tendencies or a greater tendency to break the law [36,37]. The third indication is the higher reporting of contacts when either the index case or contact was vaccinated, which may be due to the vaccinated person’s perception that contact tracing is more useful or reflects that they are more likely to comply with national guidelines [38].

Finally, the effect of residential building type on the propensity to report contacts supports the existence of infectious encounters between persons that are not identified as such. Indeed, the fact that under-reporting was higher in apartment buildings than in family houses, especially during the Omicron wave, suggests the occurrence of unperceived contagions in common areas (i.e. situations where the index case did not perceive the contact as at risk of contagion), which are more numerous and common for apartment buildings than for houses. Under-reporting was higher in buildings with more inhabitants and in buildings with shops, indicating that some contacts may go unnoticed in shared social places. These types of shared spaces, such as elevators, corridors, stairs or entrance halls, do not allow proper physical distancing and are often poorly ventilated, thus allowing potential airborne infections. The higher under-reporting in residential buildings with shops could also be due to the fact that buildings with shops are more common in urban areas with a higher population density. The absence of an effect of residential building type on under-reporting of contacts during the Alpha wave (6 January 2021–14 June 2021) can be explained by the health policies implemented during that period (private gathering restrictions, wearing face masks). This finding indicates that these public health policies reduced the number of unperceived contagions.

There are several limitations to this study. First, due to our analysis design, we restricted the study to infections occurring at the same address. As a consequence, tests results without addresses (3.4%) were removed from our analysis, leading to potential selection bias. Another potential limitation of the use of addresses is that some of the reported addresses may not correspond to the actual place of living. This type of misclassification bias may underestimate the number of secondary infections (i.e. bias towards the null). Also, our analysis does not consider contacts becoming infected at other places, and a similar analysis performed at the place of work or in different settings could be of interest. Second, the use of aggregated socioeconomic indicators at the neighbourhood level could cause ecological fallacy, where the effect observed is caused by a variable at the person level. Third, as our study is based on a positive registered test, it ignores all COVID-19-positive persons who only performed self-tests or did not test (because they did not want to, or because they were asymptomatic). Although most positive self-tests were confirmed by official registered testing, the real coverage of all secondary infections occurring at a given address is likely lower than the one reported in the present study. Finally, as with every observational study, we cannot rule out residual confounding in the multivariate analysis, although the rich register data allowed adjusting for most of the important factors.

Nevertheless, this study offers a solid estimation of the proportion of reported infectious contacts at a given address using an extensive operational register of all SARS-CoV-2 tests performed in the state of Geneva during a period covering four SARS-CoV-2 variants. The analysis based on permutations at the neighbourhood level allowed us to minimise the number of contaminations occurring at other places such as schools, grocery shops or public transportation, thus providing insights into the systemic, behavioural and living factors influencing reporting contacts. The sensitivity analysis conducted showed the robustness of our results.

## Conclusion

The overall contact coverage estimated in our study and its decrease during high COVID-19 epidemic activity periods indicates that contact tracing alone could not stop the spread of SARS-CoV-2. Contact tracing coverage could be improved by social outreach targeting populations such as those living in wealthy neighbourhoods or large residential buildings. To further reduce the propagation of SARS-CoV-2, public health authorities should consider additional non-pharmaceutical interventions aiming to avoid unperceived contagions, such as wearing face masks, cleaning and clearing the air or restricting gatherings.

## Supplementary Data

629000 Supplementary Material
