# Supplementary material for: Time trends and modifiable factors of COVID-19 contact tracing coverage, Geneva, Switzerland, June 2020 to February 2022
Source: Euro Surveill. 2024 Jan 18;29(3):2300228. doi: 10.2807/1560-7917.ES.2024.29.3.2300228 (PMC10797663; doi:10.2807/1560-7917.ES.2024.29.3.2300228)
Supplement: Supplementary Material [file 23-00228_MONGIN_Supplement.pdf]

Time trends and modifiable factors of COVID-19 contact tracing coverage, Geneva, Switzerland, June 2020 to February 2022

This supplementary material is hosted by *Eurosurveillance* as supporting information alongside the article 'Time trends and modifiable factors of COVID-19 contact tracing coverage, Geneva, Switzerland, June 2020 to February 2022', on behalf of the authors, who remain responsible for the accuracy and appropriateness of the content. The same standards for ethics, copyright, attributions and permissions as for the article apply. Supplements are not edited by *Eurosurveillance* and the journal is not responsible for the maintenance of any links or email addresses provided therein.

Table of contents

Methods 2
Covariates 2
Building types 2
CATI index 2
Definition, declaration and follow up of contacts. 3
SARS-CoV-2 variants 3
Non-pharmaceutical interventions 4
Vaccination 5
Results 6
Influence of the delay 6
Sensitivity analysis 8
Delay of 6 days between tests 8
Delay of 14 days between tests 10

Methods

Covariates

Building types

In the original dataset, we used the official classification used in Switzerland, which includes the following categories:

- "Individual houses"
- "Houses with several livings" (a category which also include buildings, but without annex usage such as shops or others)
- "Building with annex usage"
- "Building with partial use for living" (e.g. schools where there is a janitor living)
- "Building without living". This category was not considered and absent from our dataset of test results.

The dataset of all address of Geneva provided by the state of Geneva included also the number of housing at the address, and the number of people living at the address. There were ~10% missing for the official building categories and the number of housing at the address. The missing were imputed as follows:

- number of housing: as the rounded mean of the number of housing of the 4 closest address with non-missing information
- official building categories: as the predominant category in the 10 closest sites with non-missing information.

The building category was then operationalized as follow:

- house with isolated persons: any building with less or equal than 2 persons
- Houses (family houses): "Individual houses", or "Houses with several livings" with less or equal than 2 housings
- Building with no shops and less than 40 inhabitants: "Houses with several livings" with more than 2 housings or building with partial use for living and less than 40 people living at the address
- Building with no shops and more than 40 inhabitants: "Houses with several livings" with more than 2 housings or building with partial use for living and less than 40 people living at the address
- Building with shops and less than 40 inhabitants: "Building with annex usage" with between 2 and 40 persons living at the address
- Building with shops and more than 40 inhabitants: "Building with annex usage" with more than 40 persons living at the address

CATI index

Socio-economic vulnerability was assessed using a neighbourhood socio-economic vulnerability index defined by the Center for Territorial Analysis of Inequalities (CATI-GE). The state of Geneva provided for each area 6 variables corresponding to different aspects of vulnerability: the proportion of household perceiving a housing allowance, the median income of households, the share of low income, the share of students coming from a modest family, the share of active persons registered to the unemployment office, and the share of persons perceiving social subsidies (e.g., disability

subsidies, health insurance subsidies, or supplementary pension benefits). Table 1 summarizes these variables and indicates the threshold used to determine if the variable contribute the socio-economic vulnerability.

Supplementary Table 1 Composition of the neighbourhood socio-economic vulnerability index (NSVI). Each variable is computed for the geographical area.

Variable	Threshold defining a low socio-economic level in the geographical area
Proportion of housing allowance	Above the fourth quartile of the overall proportion of housing allowance
Median income	Below the first quartile of the overall distribution of median income
share of low income	Above the fourth quartile of the overall share of low income
share of students coming from a modest family	Above the fourth quartile of the overall share of student coming from a modest family
share of active persons registered to the unemployment office	Above the fourth quartile of the overall share of active persons registered to the unemployment office
share of person perceiving social subsidies	Above the fourth quartile of the overall person perceiving social subsidies

The CATI index for each area was operationalized as the sum of the six dichotomized variables (1 low socio-economic level, 0 non-low socio-economic level), resulting in a score ranging from 0 to 6. In order to have similar sizes of group, we defined four groups: the reference wealthy group of area with a CATI index equal to 0, slightly socio-economic vulnerable areas with a CATI index of 1, moderately socio-economic vulnerable areas with a CATI index between 2 and 3, and highly socio-economic vulnerable areas with a CATI index higher than 3.

Definition, declaration and follow up of contacts.

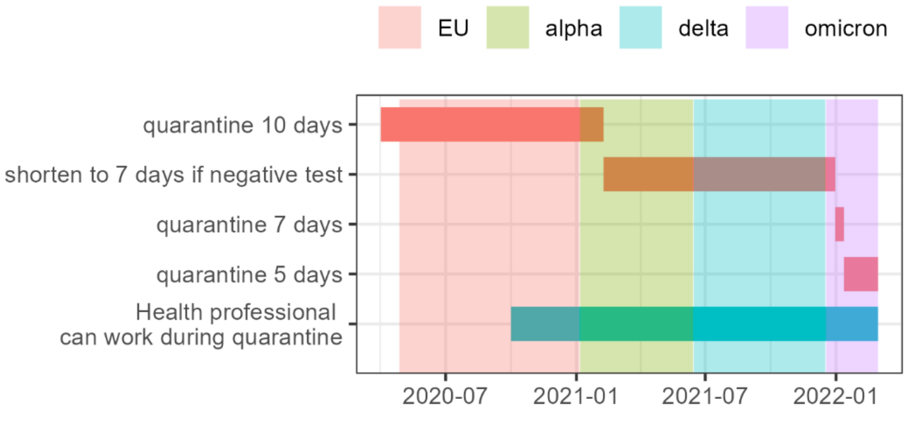

SARS-CoV-2 variants

The data used to determine the period of Variant predominance are provided by GISEAD and covariant.org, and concern the regions 1 of Switzerland, encompassing Geneva, Valais and Vaud

cantons (https://covariants.org/per-country?region=Switzerland). The data were available at the following github repository https://github.com/hodcroftlab/covariants/blob/master/cluster_tables/SwissClusters_data.json. After selecting the data for the region1 of Switzerland and the four variants of interest in our study, we modelled the VoC evolution as a rising and decreasing sigmoid in time. This approach yields the estimations presented in the graph below (estimations are solid lines, points are the experimental data)

Supplementary figure 1: share of SARS-CoV-2 Variant of Concern (VoC) in the Geneva region: measures from GISEAD (points) and the estimation based on sigmoid functions (solid lines)

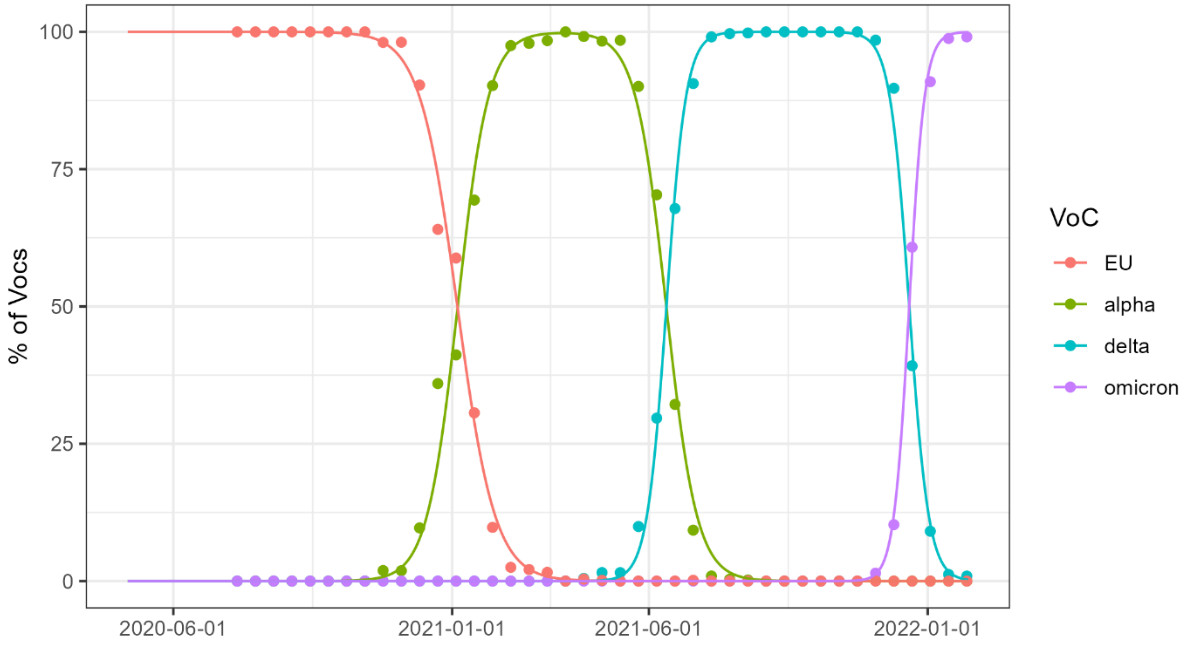

From this estimated curve, we determine the dates for two set of dominance threshold:
For 50% dominance:

VoC	EU	alpha	delta	Omicron
Start	01-06-2020	06-01-2021	15-06-2021	18-12-2021
end	05-01-2021	14-06-2021	17-12-2021	01-02-2022

Non-pharmaceutical interventions

From the first COVID-19 positive case in Geneva (26-02-2020), several non-pharmaceutical interventions (NPI) have been put in place. The list and Figure S15 below summarize the main NPIs from March 2020 to February 2022:

- From 12-03-2020 to 26-06-2021: obligation to work from home
- From 16-03-2020 to 11-05-2020: confinement, including schools closure
- From 16-03-2020 to 30-05-2020: restriction of gathering to 5 people
- From 06-07-2020 to 26-06-2021: obligation to wear a mask in public
- From 21-10-2020 to 22-03-2021: restriction of gathering to 5 people
- From 02-11-2020 to 01-03-2021: closure of non-essential shops
- From 13-09-2021 to 17-02-2022: COVID certificate is mandatory in public places
- From 29-11-2021 to 17-02-2022: obligation to wear a mask in public places

Supplementary figure 2: main non-pharmaceutical interventions (NPI) in Geneva over time. The coloured lines indicate the period during which the NPIs described on the left were active.

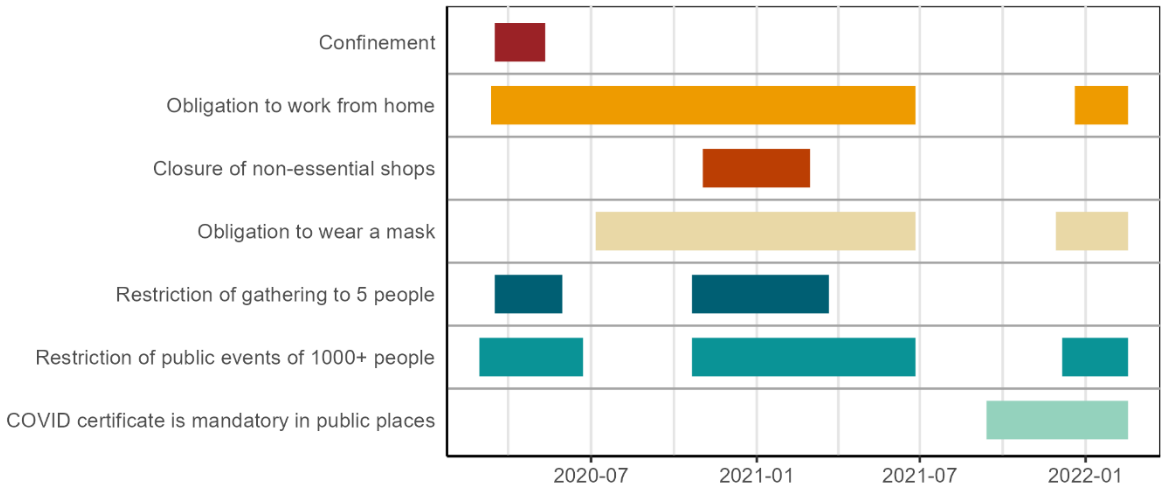

During the complete period, both PCR and antigenic tests were free of charge for the population and there were no test accessibility restrictions except for a short period during the first EU1 epidemic wave. Antigenic tests were introduced the 09-11-2020. Note that the testing capacity was saturated during the peak period of the second EU1 wave (end of October 2020) and the Omicron wave (beginning of January 2022).

Vaccination

Supplementary figure 3: Evolution in time of the proportion of the population with at least one dose in Geneva

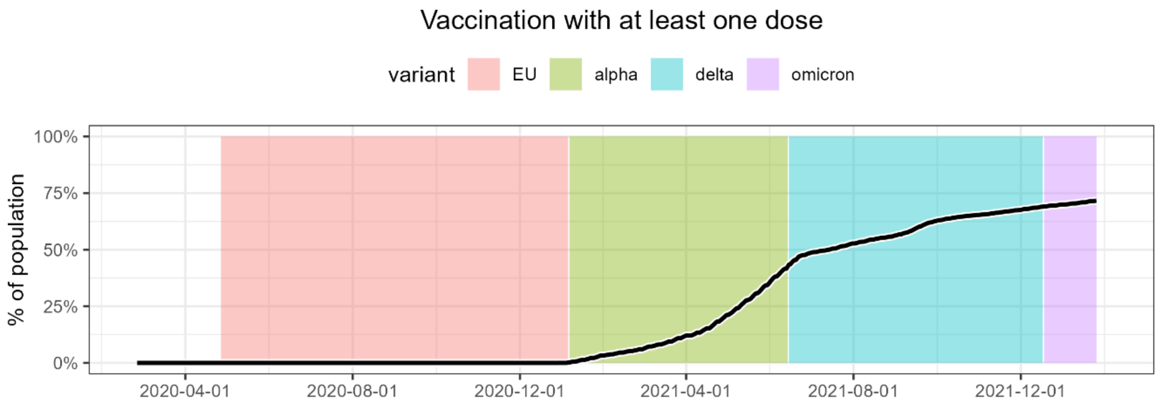

In Geneva, the first vaccination centre opened the 25-01-2021 for person aged 75 or more at first. In February, vaccination was extended to vulnerable persons under 75 (immune-compromised persons, diabetic, …) and healthcare collaborators. The targeted population was progressively extended to people over 45 years old (from 12-04-2021), people over 16 years old (from 14-05-2021) and finally to children (from 02-07-2021 and from 12-01-2022 for younger people between 5 to 11 years old respectively). The most administrated vaccine type in Geneva was the RNA-based vaccine type, such as Moderna mRNA-1273 (59.83% of the total administrated vaccine doses in Geneva) and Pfizer BNT162b2 (39.86%), other type of vaccine stands for a minor part: Janssen (0.25%) and Nuvaxovid (0.06%). The final uptake at end of February 2022 was of 71.6% of the population who received at least one dose of vaccine (time evolution in Figure S16).

Results

Influence of the delay

Supplementary figure 4: Excess of concurrent infections at the addresses as a function of the delay between the two tests. The shaded area represents the 95% CI of the estimated number of secondary infections.

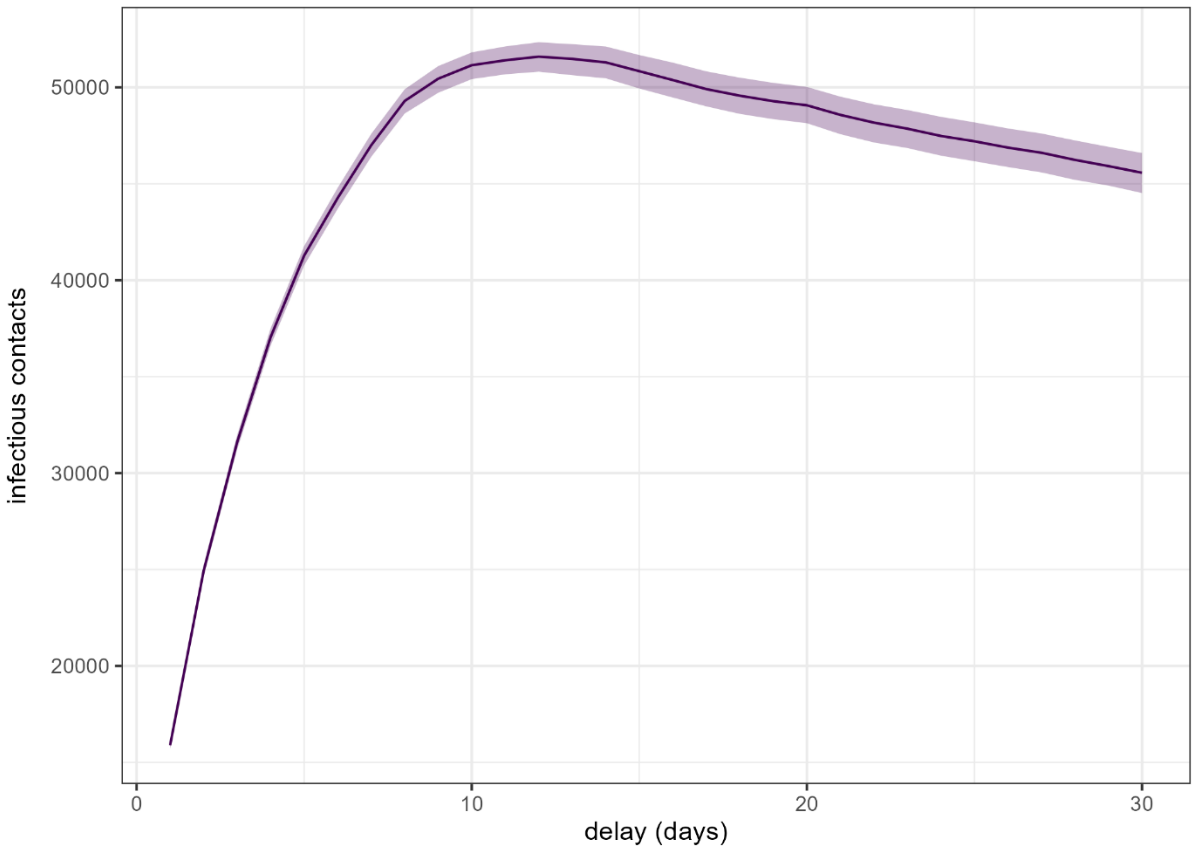

infectious contacts
50000
40000
30000
20000
0
10
20
30
delay (days)

Sensitivity analysis

Delay of 6 days between tests

Supplementary Table 2: Number of persons infected at the same address 6 days apart from each other, in the real data base, when permuting addresses on the state level (state base permutation), or on the neighbourhood level (neighbourhood base permutation). Estimations for permutation are the median of 1000 permutations given with their percentile confidence intervals (2.5% - 97.5% range).

	**total**	**EU1**	**alpha**	**delta**	**omicron**
Number of raw concurrent infections	123'304	28'574	7'297	14'888	72'545
Number of concurrent infections in permutations	78'429 CI: 79'443-77'445	15'337 CI: 15'583-15'104	1'702 CI: 1'784-1'630	6'140 CI: 6'298-5'998	55'250 CI: 55'778-54'713
Estimated contagions at the address	44'875 CI: 43'861-45'859	13'237 CI: 12'991-13'470	5'595 CI: 5'513-5'667	8'748 CI: 8'590-8'890	17'295 CI: 16'767-17'832
Number of contacts declared living at the same address	18'017	4'664	2'799	4'317	6'237
percentage of contagions declared	40.1% CI: 39.3-41.1	35.2% CI: 34.6-35.9	50% CI: 49.4-50.8	49.3% CI: 48.6-50.3	36.1% CI: 35-37.2

Supplementary Table 3: Odds ratio (OR) with their associated confidence interval of the multivariable generalized model of under-reporting of the secondary infection, when considering concurrent infection as two positive tests 6 days apart. OR with and IR not encompassing 1 are grey shaded.

Variable	EU1		alpha		delta		omicron	
	OR	95% CI	OR	95% CI	OR	95% CI	OR	95% CI
Contact age (reference: 17-65)								
0-16	1.36	0.93-1.9	1.24	0.91-1.65	1.16	0.93-1.44	1.13	0.93-1.34
65+	0.9	0.58-1.26	0.91	0.33-1.75	0.75	0.34-1.25	1.08	0.68-1.62
Index age (reference: 17-65)								
0-16	1.19	0.75-1.7	1.29	0.92-1.83	1.04	0.84-1.29	1.3	1.08-1.55
65+	0.92	0.58-1.31	0.83	0.25-1.63	0.98	0.46-1.52	0.66	0.26-1.17
Cati score (reference: 0)								
1	0.84	0.64-1.11	0.75	0.51-1.11	0.84	0.64-1.1	0.8	0.64-1
2-3	0.67	0.5-0.88	0.86	0.59-1.24	0.88	0.66-1.18	0.76	0.6-0.95
4-6	0.65	0.5-0.83	0.65	0.46-0.92	0.64	0.49-0.84	0.58	0.46-0.73
Gender (reference: men-men)								
Women-women	1.22	0.81-1.81	1.09	0.72-1.62	1.34	0.98-1.82	1.05	0.82-1.37
mixed	1.27	0.91-1.81	1.23	0.89-1.78	1.24	0.94-1.64	1.06	0.85-1.36
Type of building (single house)								
House with isolated persons	1.96	0.78-4.52	1.24	0.41-4.82	3.8	0.95-13.33	1.9	0.75-3.93
Building without shops less than 40 inhabitants	0.99	0.63-1.75	0.79	0.42-1.56	1.1	0.69-1.9	0.99	0.69-1.46
Building without shops more than 40 inhabitants	1.5	0.98-2.53	0.92	0.54-1.85	1.56	0.96-2.62	1.64	1.15-2.37
Building with shops less than 40 inhabitants	1.57	0.99-2.6	1.17	0.61-2.45	1.78	1.06-3.18	2.06	1.44-3.13
Building with shops more than 40 inhabitants	2.09	1.34-3.49	0.98	0.56-2	1.79	1.09-3.11	2.5	1.69-3.64
Immune status (both non vaccinated)								
Index : not vaccinated; contact : vaccinated			0.6	0.14-1.3	0.82	0.58-1.13	0.82	0.64-1.04
Index : vaccinated; contact : not vaccinated			0.78	0.25-1.62	0.66	0.46-0.9	0.85	0.68-1.06
Index : vaccinated; contact : vaccinated			1.78	0-10.53	1.04	0.69-1.52	0.9	0.7-1.12

Delay of 14 days between tests

Supplementary Table 4: Number of persons infected at the same address 14 days apart from each other, in the real data base, when permuting addresses on the state level (state base permutation), or on the neighbourhood level (neighbourhood base permutation). Estimations for permutation are the median of 1000 permutations given with their percentile confidence intervals (2.5% - 97.5% range).

	**total**	**EU1**	**alpha**	**delta**	**omicron**
Number of raw concurrent infections	198'868	45'667	10'585	22'425	120'191
Number of concurrent infections in permutations	149'973 CI: 151'568-148'468	28'814 CI: 29'186-28'448	3'154 CI: 3'274-3'049	12'393 CI: 12'629-12'142	105'612 CI: 106'479-104'829
Estimated contagions at the address	48'895 CI: 47'300-50'400	16'853 CI: 16'481-17'219	7'431 CI: 7'311-7'536	10'032 CI: 9'796-10'283	14'579 CI: 13'712-15'362
Number of contacts declared living at the same address	21'733	5'592	3'821	5'244	7'076
percentage of contagions declared	44.4% CI: 43.1-45.9	33.2% CI: 32.5-33.9	51.4% CI: 50.7-52.3	52.3% CI: 51-53.5	48.5% CI: 46.1-51.6

Supplementary Table 5: Odds ratio (OR) with their associated confidence interval of the multivariable generalized model of under-reporting of the secondary infection, when considering concurrent infection as two positive tests 14 days apart. OR with and IR not encompassing 1 are grey shaded.

Variable	EU1		alpha		delta		omicron	
	OR	95% CI	OR	95% CI	OR	95% CI	OR	95% CI
Contact age (reference: 17-65)								
0-16	1.25	0.94-1.65	1.16	0.89-1.47	1.18	0.98-1.43	1.07	0.89-1.29
65+	0.93	0.61-1.27	0.91	0.44-1.61	0.91	0.52-1.41	1.11	0.68-1.65
Index age (reference: 17-65)								
0-16	1.12	0.79-1.49	1.19	0.88-1.59	0.97	0.8-1.17	1.25	1.04-1.49
65+	0.93	0.64-1.3	0.89	0.41-1.55	0.94	0.53-1.4	0.71	0.3-1.2
Cati score (reference: 0)								
1	0.88	0.7-1.13	0.77	0.56-1.06	0.91	0.7-1.16	0.82	0.65-1.01
2-3	0.68	0.52-0.88	0.84	0.61-1.13	0.91	0.7-1.16	0.78	0.62-0.96
4-6	0.64	0.51-0.8	0.63	0.47-0.83	0.66	0.52-0.86	0.59	0.47-0.72
Gender (reference: men-men)								
Women-women	1.19	0.85-1.73	0.98	0.69-1.38	1.31	1.02-1.76	1.07	0.85-1.42
mixed	1.2	0.92-1.65	1.1	0.82-1.45	1.18	0.93-1.52	1.06	0.87-1.32
Type of building (single house)								
House with isolated persons	1.95	0.83-3.99	1.69	0.54-5.09	3.4	1.15-8.98	2.5	1.07-4.72
Building without shops less than 40 inhabitants	1.2	0.78-2.04	0.76	0.44-1.42	1.16	0.78-1.84	1.31	0.92-1.9
Building without shops more than 40 inhabitants	2.08	1.37-3.48	1.11	0.65-1.95	1.87	1.31-2.89	2.33	1.67-3.34
Building with shops less than 40 inhabitants	1.99	1.27-3.43	1.24	0.72-2.29	2	1.32-3.15	2.64	1.83-3.95
Building with shops more than 40 inhabitants	2.94	1.85-5.03	1.18	0.7-2.15	2.27	1.49-3.53	3.6	2.54-5.22
Immune status (both non vaccinated)								
Index : not vaccinated; contact : vaccinated			0.67	0.21-1.37	0.85	0.65-1.11	0.85	0.67-1.08
Index : vaccinated; contact : not vaccinated			0.7	0.27-1.36	0.72	0.52-0.96	0.85	0.68-1.04
Index : vaccinated; contact : vaccinated			1.45	0-5.94	0.94	0.67-1.31	0.9	0.72-1.13
